# Enhanced response inhibition in experienced fencers

**DOI:** 10.1038/srep16282

**Published:** 2015-11-06

**Authors:** Dandan Zhang, Haiyan Ding, Xiaochun Wang, Changzhu Qi, Yuejia Luo

**Affiliations:** 1 Institute of Affective and Social Neuroscience, Shenzhen University, Shenzhen, 518060, China; 2 Center for Biomedical Imaging Research, Department of Biomedical Engineering, Tsinghua University, Beijing, 100084, China; 3 School of Kinesiology, Shanghai University of Sport, Shanghai, 200438, China; 4 Research Center of Sport Psychology, Wuhan Sports University, Wuhan, 430079, China

## Abstract

The inhibition of a prepotent response is an essential executive function which enables us to suppress inappropriate actions in a given context. Individuals with fencing expertise exhibit behavioral advantages on tasks with high demands on response inhibition. This study examines the electrophysiological basis for the superior response inhibition in experienced fencers. In the Go/Nogo task where frequent stimuli required a motor response while reaction had to be withheld to rare stimuli, the fencers, compared with the non-fencers, exhibited behavioral as well as electrophysiological advantages when suppressing prepotent responses. The superior response inhibition in the fencers was characterized by enhanced Nogo-N2 and reduced Nogo-P3. Single-trial analysis revealed that the amplitude difference of the Nogo-N2 between two groups was caused by lower single-trial latency variability in the fencers (may be due to low attentional fluctuation and/or stable neural processing speed) while the amplitude difference of the Nogo-P3 resulted from truly weaker neural activity in the fencers (may be because few cognitive sources are needed and few control efforts are made). The two inhibition-related components are distinct neurophysiological indexes that, on the one hand, provide effective guidance to titrate the level of executive function in fencers, and on the other hand, facilitate to monitor fencers’ improvement in the training process.

Executive control refers to a set of top-down mental processes needed when you are engaged in situations requiring decision-making, conflict resolution, error correction, and response inhibition^1^,^2^. Among these processes, the inhibition of a prepotent response is an essential executive function in everyday life^3^,^4^. Response inhibition is associated with the ability to suppress actions that are inappropriate/unwanted in a given context and that interfere with goal-driven behavior^5^,^6^,^7^. This is the ability that makes it possible for us to choose how we react rather than being unthinking creatures of habit and impulsion^8^.

The requirement to suppress a prepotent response is most frequently studied using the Go/Nogo task^7^,^9^,^10^. In this paradigm, the subject is required to respond with high speed to Go stimuli and to refrain from responding to Nogo stimuli^11^,^12^. By having many more Go than Nogo trials, responding rather than inhibiting is made prepotent^7^. Neuropsychology and cognitive neuroscience have revealed that Nogo stimuli elicit different response-related processes from those elicited by Go stimuli (i.e., response inhibition vs. response activation)^11^. According to functional magnetic resonance imaging studies, the inferior frontal gyrus and the pre-supplementary motor area show strong activations in the Nogo condition^4^,^5^,^12^,^13^,^14^. The event-related potential (ERP) studies have reported two major components in relation to response inhibition^12^. The first is Nogo-N2, which appears 200–400 ms with a frontal-midline maximum and displays larger negative amplitudes in the Nogo condition^11^,^15^,^16^,^17^,^18^,^19^. In general, the N2 enhancement to Nogo stimuli has been interpreted as a premotor inhibitory process that suppresses the incorrect response prior to reaction stage^20^,^21^,^22^,^23^. The second component is Nogo-P3, which appears 300–600 ms with a frontocentral maximum and displays larger and later positive amplitudes in the Nogo condition^11^,^16^,^19^,^22^,^24^. Unlike the Nogo-N2 (reflecting an early inhibition of response execution), the Nogo-P3 is usually considered as a later monitoring and evaluation of inhibition process^12^,^15^,^25^,^26^.

Recent years have witnessed an unprecedented interest in understanding the mechanisms of response inhibition, since it is thought to be a core deficit in attention-deficit/hyperactivity disorder (ADHD)^6^,^7^,^27^,^28^,^29^,^30^,^31^, drug abuse and addiction^30^,^32^,^33^,^34^,^35^,^36^, alcoholics^36^,^37^,^38^,^39^,^40^,^41^, Huntington’s disease^42^, obsessive-compulsive disorder^5^,^43^, and autism spectrum disorder^44^,^45^. However, while research on these diseases suggests that deficits in response inhibition are potentially inheritable characteristics and could service as candidate endophenotypes for genetic investigation^7^,^12^,^46^,^47^,^48^, very few studies have investigated the response inhibition in experts who have special executive talents or skills in a given area (except a handful of studies^49^,^50^,^51^,^52^). The importance of characterizing the response inhibition in experts is that, on the one hand, it may open new doors for a deeper understanding of the mechanisms behind inhibitory control deficits and thus facilitate the diagnosis, treatment and prevention of relevant diseases. On the other hand, because of the apparent demand to suppress inappropriate reactions in a large number of individual and social activities, distinct neurophysiological features in experts may serve as biomarkers with the capacity to measure the level of executive function and assess individuals’ potentials in special careers, such as open-skill sport, special troop, interpreter, and pilot.

Contemporary fencing is an open-skill sport which requires fencers to quickly switch their states between offence and defense based on the judgment on whether the opponents offer an attack (a thrust with the purpose of landing a touch upon the opponent’s valid area) or a feint (a thrust with the purpose of provoking a reaction from the opponent). Individuals with fencing expertise exhibit behavioral advantages on tasks with high demands on executive functioning, particularly inhibitory control^49^,^50^,^53^, but the neural correlates of the enhanced inhibition of a prepotent response are unclear. In this study, ERP technique is employed together with the Go/Nogo task to elucidate the electrophysiological basis for the superior executive function of response inhibition in experienced fencers (compared with demographically matched non-fencers). It is expected that the Nogo-N2 and the Nogo-P3, as well as behavioral measures would show significant differences between fencers and non-fencers.

In addition to stimulus-locked averaging measures, single-trial analysis of ERP^54^,^55^,^56^,^57^ is used to investigate the trial-to-trial variations with regard to the amplitude and the latency of the Nogo-N2/P3 components. As illustrated in the study of Zhang *et al.*^58^, a larger amplitude in average N2 or P3 in one subject group may reflect truly stronger neural activity (more neural networks are allocated for inhibitory control) as compared with another group (Fig. 1A); or it may be due to the ERP responses in one group that show a more consistent latency from trial to trial (i.e. a smaller fluctuation in neural processing speed during the Go/Nogo task) (Fig. 1B). Therefore, ERP analysis on the single-trial level is essential to clarify the neural mechanisms underlying the different amplitudes of the average ERPs between fencers and non-fencers.

## Method

### Participants

Twenty-six healthy adults (14 females; age range = 18 to 22 years) and twenty-six fencers (13 females; age range = 19 to 23 years) were recruited from Shanghai University of Sport as paid participants. All the fencers had an experience in fencing (foil) for at least six years and had won at least one national/international championship. The subjects in control group were age- and education-matched non-athletes. All participants were right-handed and had normal or corrected-to-normal vision. They gave their written informed consent prior to the experiment. The experimental protocol was approved by the local ethics committee (Shanghai University of Sport) and this study was performed strictly in accordance with the approved guidelines.

### Stimuli

The fixation was a cross of 0.3° × 0.3° visual angle in the center of the computer monitor. The stimuli consisted of vertical and horizontal white bars on the black background (four pictures with a visual angle of 4° × 4°; Fig. 2). The four configurations were displayed randomly with equal probability within Go condition (*p* = 0.2, i.e., *p* = 0.1 for each Go picture) and within Nogo condition (*p* = 0.8, i.e., *p* = 0.4 for each Nogo picture). We used two stimuli in each condition to prevent the ceiling effect.

### Procedure

Participants were seated in a dimly lit and sound-attenuated room. Stimuli were presented on a LCD monitor at a viewing distance of approximately 100 cm. Stimulus display and behavioral data acquisition were conducted using E-Prime 2.0 (Psychology Software Tools, Inc., Pittsburgh, PA).

Each trial started with a 200-to-300-ms fixation, followed by targets (Go stimuli) and non-targets (Nogo stimuli) that were presented for 200 ms. A black blank screen appeared after the stimulus and lasted for 1500 ms. Participants were required to press a button with their right index finger on the response box as quick as possible when the Go stimuli appeared, while they should withhold the behavioral responses when the Nogo stimuli appeared. The Go and Nogo trials were presented in random order with a probability of 4:1. The total experiment consisted of four blocks, with 100 trials in each block.

### EEG recording and analysis

Brain electrical activity was recorded referentially against left mastoid and off-line re-referenced to the average of the left and right mastoids, by a 32-channel amplifier with a sampling frequency of 250 Hz (Brain Products, Gilching, Germany). All the electrooculogram and electroencephalogram (EEG) electrodes worked with electrode impedances kept below 5 kΩ. Ocular artifacts were removed from EEGs using a regression procedure implemented in NeuroScan software (Scan 4.3; NeuroScan Inc., Herndon, USA).

The data analysis and result display in this study were performed using Matlab R2011a (MathWorks, Natick, USA). The recorded EEG data were filtered with a 0.01–30 Hz finite impulse response filter with zero phase distortion. Filtered data were segmented beginning 200 ms prior to the onset of stimulus and lasting for 1200 ms. All epochs were baseline-corrected with respect to the mean voltage over the 200 ms preceding the onset of stimulus, followed by averaging in association with experimental conditions.

This study focused on the ERP components associated with inhibitory control. In particular, we analyzed the peak latency and peak amplitude of frontal N2, fronto-central P3 (Nogo), and parietal P3 (Go) between Go and Nogo conditions and between fencers and non-fencers. The ERP amplitudes were measured using different sets of electrodes in accordance with grand-mean ERP topographies and relevant literatures^11^,^15^,^16^,^17^,^18^,^19^,^22^,^24^. Accordingly, the mean of the peak amplitude of the N2 was calculated at the electrode sites of Fz, FC1 and FC2, with its peak detected within a time window of 200–400 ms after stimulus onset. The mean of the peak amplitude of the P3 was calculated at the electrode sites of Cz, FC1 and FC2 (Nogo condition) or at the electrode sites of Pz, CP1 and CP2 (Go condition), with its peak detected within a time window of 300–600 ms (Nogo condition) or 200–500 ms (Go condition) after stimulus onset.

Single-trial peak detection of N2 and P3 was performed on the average ERP waveforms computed based on the same electrode sets as in the average ERP analysis. The Maximum Likelihood Estimation (MLE) technique was employed to detect the occurrence of the component peak on the single-trial basis^59^. The MLE assumes that the ERP signal that hides in EEG background activity has an invariant shape but may vary both in its latency and amplitude. By maximizing the log likelihood function of the model in the frequency domain, the MLE estimates the unknown parameters of ERP signals such as latencies and amplitudes in single trials. Assuming that peak amplitude and peak latency across trials obey normal distributions^60^, the peak amplitude of the average ERP is primarily influenced by two variables, namely, the mean of the single-trial peak amplitude and the standard deviation of the single-trial peak latency, both of which are measured to characterized the peak amplitude of the average ERP in this study.

### Statistics

Statistical analysis was performed using SPSS Statistics 20.0 (IBM, Somers, USA). Descriptive data were presented as mean ± standard error. The significance level was set at 0.05.

Two-way repeated-measures ANOVAs were performed on measurements of the behavioral and ERP data, with response assignment (Go and Nogo) as the within-subject factor, and group (fencer and non-fencer) as the between-subject factor. Two-way repeated-measures ANOVA were also performed on the P3 peak amplitudes, with response assignment and electrode site (Fz, Cz and Pz) as the within-subject factors^11^. Greenhouse-Geisser correction for ANOVA tests was used whenever appropriate. Post-hoc testing of significant main effects was conducted using Bonferroni method. Significant interactions were analyzed using simple effects model. Partial eta-squared (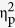) was reported to demonstrate the effect size in ANOVA tests.

To counterbalance the number of trials between Go and Nogo conditions, only 1/4 Nogo trials were randomly selected and used to statistically compare with Go trials and plot the ERP waveforms in the study.

## Results

For the sake of brevity, this section only reports the most important results. Please refer to the supplementary material for the other significant findings.

### Accuracy rate (ACC)

The interaction effect of response assignment by group was significant (*F*(1,50) = 4.80; *p* = 0.033; 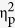 = 0.088). Simple effect analysis indicated that the ACC in the Nogo trials was higher in the fencers (0.421 ± 0.033) compared with that in the non-fencers (0.310 ± 0.033) (*F*(1,50) = 5.51; *p* = 0.023). The ACC in the Go trials did not differ between groups (*F*(1,50) < 1; fencer = 0.989 ± 0.004; non-fencer = 0.983 ± 0.004).

### Reaction time (RT)

The main effect of group was significant (*F*(1,50) = 6.30; *p* = 0.015; 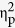 = 0.112): the RT in the fencers (710 ± 20 ms) was shorter than that in the non-fencers (780 ± 20 ms).

### Average measures of the N2

The interaction effect of response assignment by group was significant on the peak amplitude of the average N2 (*F*(1,50) = 4.18; *p* = 0.046; 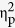 = 0.077; Fig. 3). The N2 amplitude in the Nogo condition (*F*(1,50) = 8.75; *p* = 0.005) was larger in the fencers (−3.97 ± 0.45 μV) compared with the non-fencers (−2.07 ± 0.45 μV), whereas the group effect was not significant in the Go condition (*F*(1,50) < 1; fencer = 0.74 ± 0.45 μV; non-fencer = 0.82 ± 0.45 μV).

The interaction effect of response assignment by group was significant on the peak latency of the average N2 (*F*(1,50) = 6.42; *p* = 0.014; 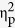 = 0.114; Fig. 3). The N2 latency in the Nogo condition (*F*(1,50) = 8.90; *p* = 0.004) was shorter in the fencers (280 ± 8.09 ms) compared with the non-fencers (314 ± 8.09 ms), while the group effect was not significant in the Go condition (*F*(1,50) < 1; fencer = 244 ± 7.80 ms; non-fencer = 236 ± 7.80 ms).

### Single-trial measures of the N2

The interaction effect of response assignment by group was significant on the standard deviation of the single-trial peak latency (*F*(1,50) = 5.39; *p* = 0.024; 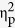 = 0.097). The N2 latency jitter in the Nogo condition (*F*(1,50) = 11.3; *p* = 0.001) was smaller in the fencers (standard deviation of latency = 21.6 ± 0.46 ms) compared with the non-fencers (23.8 ± 0.46 ms), while the group effect was not significant in the Go condition (*F*(1,50) < 1; fencer = 25.7 ± 0.55 ms; non-fencer = 25.5 ± 0.55 ms).

No significant difference was found in the mean of the single-trial peak amplitude of the N2 across conditions or groups.

### Average measures of the P3

The interaction effect of response assignment by group was significant on the peak amplitude of the average P3 (*F*(1,50) = 5.74; *p* = 0.020; 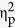 = 0.103; Figs 4 and 5). The P3 amplitude in the Nogo condition (*F*(1,50) = 5.95; *p* = 0.018) was larger in the non-fencers (10.4 ± 0.64 μV) compared with the fencers (8.24 ± 0.64 μV) while the group effect was not significant in the Go condition (*F*(1,50) < 1; fencer = 6.53 ± 0.37 μV; non-fencer = 6.95 ± 0.37 μV).

The interaction effect of response assignment by group was significant on the peak latency of the average P3 (*F*(1,50) = 4.47; *p* = 0.040; 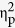 = 0.082; Figs 4 and 5). The P3 latency in the Nogo condition (*F*(1,50) = 6.81; *p* = 0.012) was shorter in the fencers (482 ± 11.8 ms) compared with the non-fencers (526 ± 11.8 ms), while the group effect was not significant in the Go condition (*F*(1,50) < 1; fencer = 355 ± 9.93 ms; non-fencer = 356 ± 9.93 ms).

Then the peak amplitude of the average P3 was tested for its topographic difference between Go and Nogo conditions. The interaction effect of response assignment by electrode site was significant (*F*(2,102) = 5.87; *p* = 0.006; 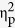 = 0.103; Figs 3, 4, 5). The P3 amplitude in the Go condition (*F*(2,102) = 9.46; *p* < 0.001) was largest at Pz (6.74 ± 0.26 μV) (Cz = 5.50 ± 0.55 μV; Fz = 4.34 ± 0.33 μV; Fz *vs*. Pz, *p* < 0.001). However, the P3 amplitude in the Nogo condition (*F*(2,102) = 18.2; *p* < 0.001) showed a different topographic pattern: it was largest at Cz (9.34 ± 0.48 μV) (Fz = 5.63 ± 0.46 μV; Pz = 7.95 ± 0.50 μV; Cz *vs*. Pz, *p* = 0.002; Fz *vs*. Cz, *p* < 0.001).

### Single-trial measures of the P3

The interaction effect of response assignment by group was significant on the mean of the single-trial P3 peak amplitude (*F*(1,50) = 4.68; *p* = 0.035; 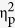 = 0.086). The P3 amplitude in the Nogo condition (*F*(1,50) = 4.74; *p* = 0.034) was larger in the non-fencers (13.9 ± 0.87 μV) compared with the fencers (11.2 ± 0.87 μV) while the group effect was not significant in the Go condition (*F*(1,50) < 1; fencer = 8.97 ± 0.50 μV; non-fencer = 9.36 ± 0.50 μV).

No significant difference was found in the standard deviation of the single-trial peak latency of the P3 across conditions or groups.

## Discussion

Cancelling initiated responses timely is especially crucial and frequently happened during fencing practice (e.g., in the case when a feint is provided by the opponent). The present study employs ERPs to examine the neural correlates for fencers’ inhibition advantage according to the hypothesis that experienced fencers have enhanced executive function of response inhibition^49^,^50^,^53^.

### Behavioral data

Previous behavioral studies in patients indicated a deterioration of Go/Nogo performance caused by an impaired ability to inhibit unwanted behavior. For example, individuals diagnosed with Huntington’s disease showed more false alarms in the Nogo condition^42^; patients with Tourette’s syndrome had longer RTs than healthy controls in Go trials and made more errors in total^61^; and obese children exhibited lower response accuracy relative to healthy weight children in the Nogo condition^62^. We found in the current study that the number of mistaken responses the fencers made on Nogo trials (false alarm) was significantly smaller than that made by the non-fencers. At the same time, the average RT to Go as well as to Nogo trials was significantly shorter in the fencers compared with the non-fencers. Similarly, using the Go/Nogo paradigm, Chan *et al.*^49^ observed that high-fit fencers committed significantly fewer errors compared to the non-fencers; and Kida *et al.*^52^ showed that the Go/Nogo RT for baseball players was significantly shorter than that of the non-athletes. The current study suggests that open-skill athletes are superior to non-athletes in both ACC and RT in the Go/Nogo task.

### ERP data

It was observed in our data that the average Nogo-N2 elicited in the fencers was larger and with an earlier latency compared with that elicited in the non-fencers. The Nogo-N2 is known to reflect an inhibition of response execution^20^,^21^,^23^, and this inhibitory process is usually located at the pre-motor rather than at the motor level^22^. Previous Go/Nogo studies in healthy adults found that a smaller and later Nogo-N2 was evoked in subjects with a high false alarm rate compared with those with a low false alarm rate^22^. Similarly, studies in patients indicated that Nogo-N2 amplitudes were negatively correlated with the number of symptoms in ADHD^20^,^27^; and the N2 enhancement to Nogo trials was strongly reduced in the depressed individuals^63^. The current finding that Nogo stimuli elicited larger and earlier N2 in the fencers is consistent with previous studies, which suggested that the improvement of inhibitory control is accompanied by a larger and/or earlier Nogo-N2^50^,^51^.

**Figure 1:**
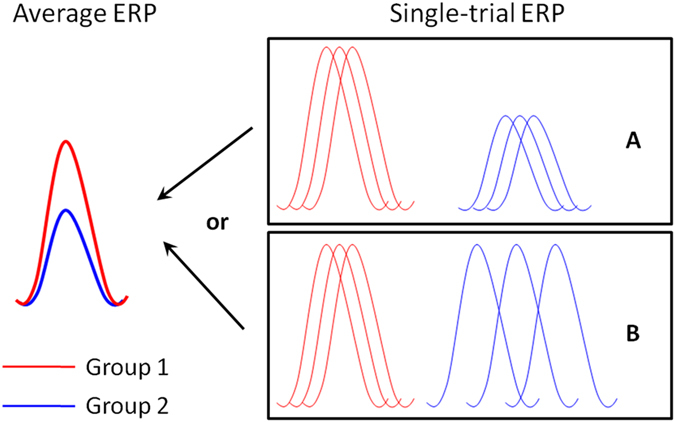
Illustration of two potential mechanisms for amplitude differences in average ERP. The average amplitude difference between fencers and non-fencers may due to (**A**) true amplitude difference in single trials, or (**B**) the difference in single-trial latency variability.

**Figure 2:**
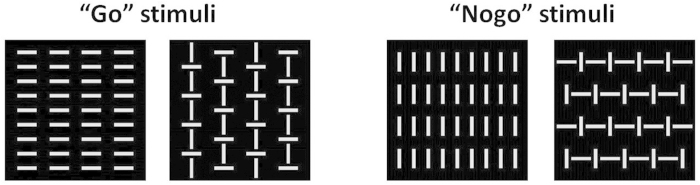
The stimuli and their response assignments in the experiment. The stimuli consisted of vertical and horizontal white bars. The Nogo stimuli were made by rotating the bars in the Go stimuli by 90°.

**Figure 3:**
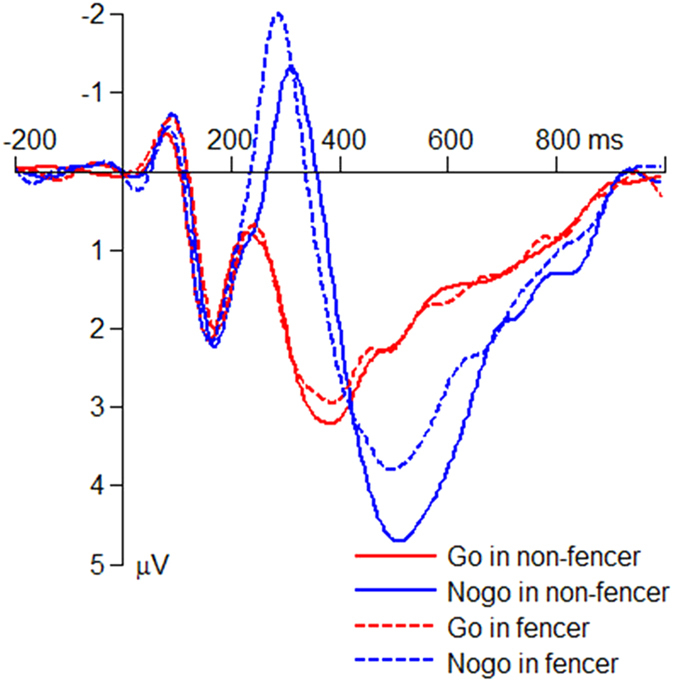
The grand-mean ERP waveforms in the Go and the Nogo conditions at the electrode site of Fz. All the Go trials and 25% Nogo trials (randomly selected from the data) were averaged to produce the grand-mean waveforms.

**Figure 4:**
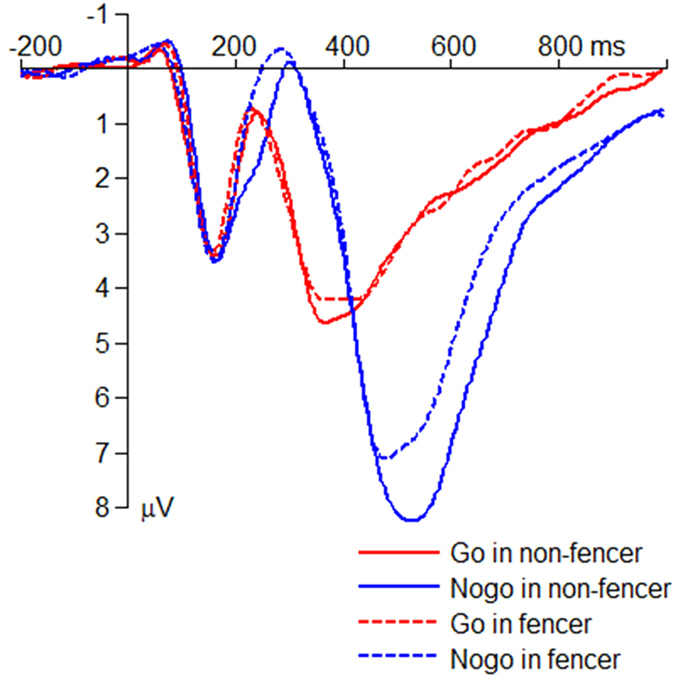
The grand-mean ERP waveforms in the Go and the Nogo conditions at the electrode site of Cz.

**Figure 5:**
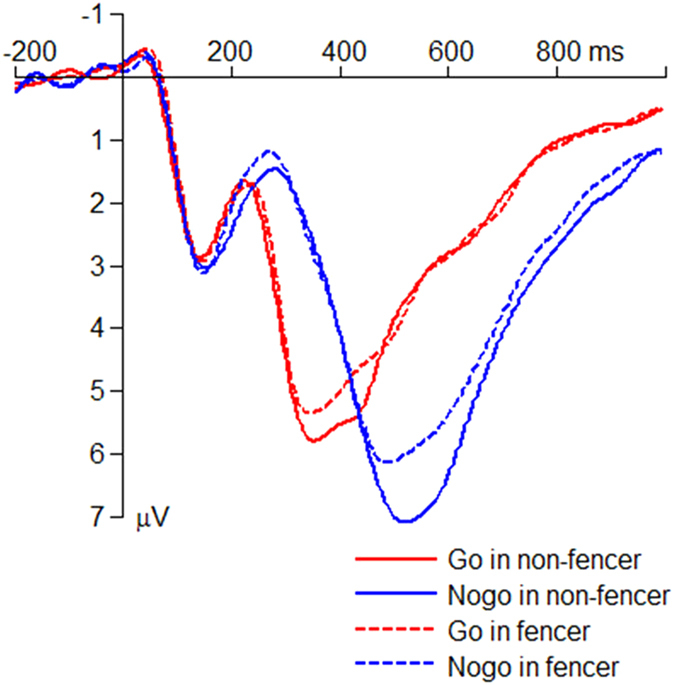
The grand-mean ERP waveforms in the Go and the Nogo conditions at the electrode site of Pz.

More importantly, this study examined the single-trial characteristics of the N2. Our result suggested that the enhanced Nogo-N2 in the fencers was due to smaller latency fluctuations on the trial-to-trial basis in this group (Fig. 1B). A potential explanation for the less variability in the Nogo-N2 latency is that fencers, compared with non-fencers, may have fewer attentional fluctuations and/or have a more stable neural processing speed when inhibition control is needed (see the study of Ford *et al.*^57^ for example), thus producing more consistent single-trial latencies at the early stage of response inhibition. This interpretation is in harmony with Rentrop *et al.*^64^, who observed in the Go/Nogo task that patients with schizophrenia displayed an increased variability in N2 latency, indicating a temporal instability of information processing associated with the disease.

Another interesting finding of this study was that the average Nogo-P3 differed significantly between groups: compared to the non-fencers, the fencers displayed an earlier and amplitude-reduced P3 in the Nogo condition. The Nogo-P3 is usually considered as the evaluation and monitoring of inhibition process^12^,^15^,^25^,^26^,^65^. Researchers have found that heavy drinkers showed significantly greater activity in the inhibitory control regions on the Nogo trials and displayed significantly larger NoGo-P3 amplitudes than controls; this result suggested that heavy drinkers may have impaired neural functions related to response inhibition so their brain needs to allocate more cognitive sources and make more control efforts to successfully withhold a response^40^,^41^. Similarly, Taddei *et al.*^50^ found that age-related compensatory neural responses occur during response inhibition, reflected by a delayed Nogo-P3 in older subjects compared with younger subjects. According to these results, we propose that the group difference in the Nogo-P3 of the current study indicates that fencing expertise improves the inhibitory function of individuals and makes their brain able to evaluate and monitor the inhibition of a prepotent response in a more efficient manner. Furthermore, single-trial peak estimates of the P3 indicated that the expertise-induced inhibitory improvement was reflected by true amplitude reductions in single trials (i.e. fewer neural resources were needed in response inhibition for experienced fencers) (Fig. 1A). It is known that P3 amplitude reflects demands on “perceptual-central” resources and is closely related to the intensity of processing^66^. The real P3 amplitude reduction observed in single trials provides strong evidence that long-term training in fencing significantly enhances the neural efficiency of inhibition processing, resulting in a compaction of associated cognitive sources and a minimization of control efforts in the inhibitory task.

### Conclusion remarks

The current study investigated the effect of fencing expertise on the executive function of response inhibition. In the Go/Nogo task where frequent (80%) stimuli required a motor response while reaction had to be withheld to rare (20%) stimuli, the fencers, compared with the non-fencers, exhibited behavioral as well as electrophysiological advantages when suppressing prepotent responses. The superior response inhibition in the fencers was characterized by enhanced Nogo-N2 (may be due to low attentional fluctuation and stable neural processing speed in response inhibition process) and reduced Nogo-P3 (may be because few cognitive sources are allocated and few control efforts are made). These two inhibition-related ERP components are distinct neurophysiological indexes that may, on the one hand, provide effective guidance to titrate the level of executive function in fencers, and on the other hand, facilitate to monitor fencers’ improvement in the training process. Furthermore, in line with the single-trial findings, we infer that diseases relevant to inhibitory control deficits, such as ADHD and drug abuse, are likely accompanied by instability of information processing and excessive demand for neural networks during response inhibition.

## Additional Information

**How to cite this article**: Zhang, D. *et al.* Enhanced response inhibition in experienced fencers. *Sci. Rep.* **5**, 16282; doi: 10.1038/srep16282 (2015).

## Supplementary Material

Supplementary Information

## References

[b1] Baddeley A. & Della Sala S. Working memory and executive control. Philos Trans R Soc Lond B Biol Sci 351, 1397–1404 (1996). 8941951 10.1098/rstb.1996.0123

[b2] Miller E. K. & Cohen J. D. An integrative theory of prefrontal cortex function. Annu Rev Neurosci 24, 167–202 (2001). 11283309 10.1146/annurev.neuro.24.1.167

[b3] Aron A. R. The neural basis of inhibition in cognitive control. Neuroscientist 13, 214–228 (2007). 17519365 10.1177/1073858407299288

[b4] Chikazoe J. Localizing performance of go/no-go tasks to prefrontal cortical subregions. Curr Opin Psychiatry 23, 267–272 (2010). 20308899 10.1097/YCO.0b013e3283387a9f

[b5] Chambers C. D, Garavan H. & Bellgrove M. A. Insights into the neural basis of response inhibition from cognitive and clinical neuroscience. Neurosci Biobehav Rev 33, 631–646 (2009). 18835296 10.1016/j.neubiorev.2008.08.016

[b6] Mostofsky S. H. & Simmonds D. J. Response inhibition and response selection: two sides of the same coin. J Cogn Neurosci 20, 751–761 (2008). 18201122 10.1162/jocn.2008.20500

[b7] Aron A. R. & Poldrack R. A. The cognitive neuroscience of response inhibition: relevance for genetic research in attention-deficit/hyperactivity disorder. Biol Psychiatry 57, 1285–1292 (2005). 15950000 10.1016/j.biopsych.2004.10.026

[b8] Diamond A. Executive functions. Annu Rev Psychol 64, 135–168 (2013). 23020641 10.1146/annurev-psych-113011-143750 PMC4084861

[b9] Aron A. R. Introducing a special issue on stopping action and cognition. Neurosci Biobehav Rev 33, 611–612 (2009). 18977244 10.1016/j.neubiorev.2008.10.003

[b10] Bari A. & Robbins T. W. Inhibition and impulsivity: behavioral and neural basis of response control. Prog Neurobiol 108, 44–79 (2013). 23856628 10.1016/j.pneurobio.2013.06.005

[b11] Eimer M. Effects of attention and stimulus probability on ERPs in a Go/Nogo task. Biol Psychol 35, 123–138 (1993). 8507742 10.1016/0301-0511(93)90009-w

[b12] Huster R. J., Enriquez-Geppert S., Lavallee C. F., Falkenstein M. & Herrmann C. S. Electroencephalography of response inhibition tasks: functional networks and cognitive contributions. Int J Psychophysiol 87, 217–233 (2013). 22906815 10.1016/j.ijpsycho.2012.08.001

[b13] Aron A. R. From reactive to proactive and selective control: developing a richer model for stopping inappropriate responses. Biol Psychiatry 69, e55–e68 (2010). 20932513 10.1016/j.biopsych.2010.07.024 PMC3039712

[b14] Sharp D. J. *et al.* Distinct frontal systems for response inhibition, attentional capture, and error processing. Proc Natl Acad Sci USA 107, 6106–6111 (2010). 20220100 10.1073/pnas.1000175107 PMC2851908

[b15] Pfefferbaum A., Ford J. M., Weller B. J. & Kopell B. S. ERPs to response production and inhibition. Electroencephalogr Clin Neurophysiol 60, 423–434 (1985). 2580694 10.1016/0013-4694(85)91017-x

[b16] Kopp B., Mattler U., Goertz R. & Rist F. N2, P3 and the lateralized readiness potential in a nogo task involving selective response priming. Electroencephalogr Clin Neurophysiol 99, 19–27 (1996). 8758967 10.1016/0921-884x(96)95617-9

[b17] Jodo E. & Kayama Y. Relation of a negative ERP component to response inhibition in a Go/Nogo task. Electroencephalogr Clin Neurophysiol 82, 477–482 (1992). 1375556 10.1016/0013-4694(92)90054-l

[b18] Thorpe S., Fize D. & Marlot C. Speed of processing in the human visual system. Nature 381, 520–522 (1996). 8632824 10.1038/381520a0

[b19] Kok A. Effects of degradation of visual stimulation on components of the event-related potential (ERP) in go/nogo reaction tasks. Biol Psychol 23, 21–38 (1986). 3790646 10.1016/0301-0511(86)90087-6

[b20] Pliszka S. R, Liotti M. & Woldorff M. G. Inhibitory control in children with attention-deficit/hyperactivity disorder: event-related potentials identify the processing component and timing of an impaired right-frontal response-inhibition mechanism. Biol Psychiatry 48, 238–246 (2000). 10924667 10.1016/s0006-3223(00)00890-8

[b21] Geczy I., Czigler I. & Balazs L. Effects of cue information on response production and inhibition measured by event-related potentials. Acta Physiol Hung 86, 37–44 (1999). 10755168

[b22] Falkenstein M., Hoormann J. & Hohnsbein J. ERP components in Go/Nogo tasks and their relation to inhibition. Acta Psychol 101, 267–291 (1999). 10.1016/s0001-6918(99)00008-6 10344188

[b23] Roche R. A., Garavan H., Foxe J. J. & O’Mara S. M. Individual differences discriminate event-related potentials but not performance during response inhibition. Exp Brain Res 160, 60–70 (2005). 15480606 10.1007/s00221-004-1985-z

[b24] Simson R., Vaughan H. G. & Ritter W. The scalp topography of potentials in auditory and visual Go/NoGo tasks. Electroenceph Clin Neurophysiol 43, 864–875 (1977). 73454 10.1016/0013-4694(77)90009-8

[b25] Schmajuk M., Liotti M., Busse L. & Woldorff M. G. Electrophysiological activity underlying inhibitory control processes in normal adults. Neuropsychologia 44, 384–395 (2006). 16095637 10.1016/j.neuropsychologia.2005.06.005

[b26] Bokura H., Yamaguchi S. & Kobayashi S. Electrophysiological correlates for response inhibition in a Go/NoGo task. Clin Neurophysiol 112, 2224–2232 (2001). 11738192 10.1016/s1388-2457(01)00691-5

[b27] Woltering S., Liu Z., Rokeach A. & Tannock R. Neurophysiological differences in inhibitory control between adults with ADHD and their peers. Neuropsychologia 51, 1888–1895 (2013). 23831207 10.1016/j.neuropsychologia.2013.06.023

[b28] Chamberlain S. R. & Sahakian B. J. The neuropsychiatry of impulsivity. Curr Opin Psychiatr 20, 255–261 (2007). 10.1097/YCO.0b013e3280ba4989 17415079

[b29] Slaats-Willemse D., Swaab-Barneveld H., de Sonneville L., van der Meulen E. & Buitelaar J. Deficient response inhibition as a cognitive endophenotype of ADHD. J Am Acad Child Adolesc Psychiatry 42, 1242–1248 (2003). 14560175 10.1097/00004583-200310000-00016

[b30] Pattij T. & Vanderschuren L.J. The neuropharmacology of impulsive behaviour. Trends Pharmacol Sci 29, 192–199 (2008). 18304658 10.1016/j.tips.2008.01.002

[b31] Groman S. M., James A. S. & Jentsch J. D. Poor response inhibition: at the nexus between substance abuse and attention deficit/hyperactivity disorder. Neurosci Biobehav Rev 33, 690–698 (2009). 18789354 10.1016/j.neubiorev.2008.08.008 PMC2728075

[b32] Belin D., Mar A. C., Dalley J. W., Robbins T. W. & Everitt B. J. High impulsivity predicts the switch to compulsive cocaine-taking. Science 320, 1352–1355 (2008). 18535246 10.1126/science.1158136 PMC2478705

[b33] Dalley J. W. *et al.* Nucleus accumbens D2/3 receptors predict trait impulsivity and cocaine reinforcement. Science 315, 1267–1270 (2007). 17332411 10.1126/science.1137073 PMC1892797

[b34] Volkow N. D., Fowler J. S., Wang G. J. & Swanson J. M. Dopamine in drug abuse and addiction: results from imaging studies and treatment implications. Mol Psychiatry 9, 557–569 (2004). 15098002 10.1038/sj.mp.4001507

[b35] Roberts G. M. & Garavan H. Evidence of increased activation underlying cognitive control in ecstasy and cannabis users. Neuroimage 52, 429–435 (2010). 20417713 10.1016/j.neuroimage.2010.04.192

[b36] Nigg J. T. *et al.* Poor response inhibition as a predictor of problem drinking and illicit drug use in adolescents at risk for alcoholism and other substance use disorders. J Am Acad Child Adolesc Psychiatry 45, 468–475 (2006). 16601652 10.1097/01.chi.0000199028.76452.a9

[b37] Rubio G. *et al.* The role of behavioral impulsivity in the development of alcohol dependence: a 4-year follow-up study. Alcohol Clin Exp Res 32, 1681–1687 (2008). 18631324 10.1111/j.1530-0277.2008.00746.x

[b38] Pandey A. K. *et al.* Neurocognitive deficits in male alcoholics: an ERP/sLORETA analysis of the N2 component in an equal probability Go/NoGo task. Biol Psychol 89, 170–182 (2012). 22024409 10.1016/j.biopsycho.2011.10.009 PMC3245806

[b39] Kamarajan C. *et al.* Alcoholism is a disinhibitory disorder: neurophysiological evidence from a Go/No-Go task. Biol Psychol 69, 353–373 (2005). 15925035 10.1016/j.biopsycho.2004.08.004 PMC3758477

[b40] Ames S. L. *et al.* Neural correlates of a Go/NoGo task with alcohol stimuli in light and heavy young drinkers. Behav Brain Res 274, 382–389 (2014). 25172182 10.1016/j.bbr.2014.08.039 PMC4179865

[b41] López-Caneda E., Rodríguez-Holguín S., Corral M., Doallo S. & Cadaveira F. Evolution of the binge drinking pattern in college students: neurophysiological correlates. Alcohol 48, 407–418 (2014). 24835220 10.1016/j.alcohol.2014.01.009

[b42] Beste C., Saft C., Andrich J., Gold R. & Falkenstein M. Response inhibition in Huntington’s disease—A study using ERPs and sLoreta. Neuropsychologia 46, 1290–1297 (2008). 18241897 10.1016/j.neuropsychologia.2007.12.008

[b43] van Velzen L. S., Vriend C., de Wit S.J. & van den Heuvel O. A. Response inhibition and interference control in obsessive-compulsive spectrum disorders. Front Hum Neurosci 8, 419 (2014). 24966828 10.3389/fnhum.2014.00419 PMC4052433

[b44] Langen M. *et al.* Fronto-striatal circuitry and inhibitory control in autism: findings from diffusion tensor imaging tractography. Cortex 48, 183–193 (2012). 21718979 10.1016/j.cortex.2011.05.018

[b45] Duerden E.G. *et al.* Neural correlates of inhibition of socially relevant stimuli in adults with autism spectrum disorder. Brain Res 1533, 80–90 (2013). 23962468 10.1016/j.brainres.2013.08.021

[b46] Chamberlain S. R. *et al.* Impaired cognitive flexibility and motor inhibition in unaffected first-degree relatives of patients with obsessive-compulsive disorder. Am J Psychiatry 164, 335–338 (2007). 17267798 10.1176/appi.ajp.164.2.335 PMC1892796

[b47] Ersche K. D. *et al.* Abnormal brain structure implicated in stimulant drug addiction. Science 335, 601–604 (2012). 22301321 10.1126/science.1214463

[b48] Vink M., Ramsey N. F., Raemaekers M. & Kahn R. S. Striatal dysfunction in schizophrenia and unaffected relatives. Biol Psychiatry 60, 32–39 (2006). 16603134 10.1016/j.biopsych.2005.11.026

[b49] Chan J., Wong A., Liu Y., Yu J. & Yan J. Fencing expertise and physical fitness enhance action inhibition. Psychol Sport Exerc 12, 509–514 (2011).

[b50] Taddei F., Bultrini A., Spinelli D. & Di Russo F. Neural correlates of attentional and executive processing in middle-age fencers. Med Sci Sports Exerc 44, 1057–1066 (2012). 22157879 10.1249/MSS.0b013e31824529c2

[b51] Moreno S., Wodniecka Z., Tays W., Alain C. & Bialystok E. Inhibitory control in bilinguals and musicians: event related potential (ERP) evidence for experience-specific effects. PLoS One 9, e94169 (2014). 24743321 10.1371/journal.pone.0094169 PMC3990547

[b52] Kida N., Oda S. & Matsumura M. Intensive baseball practice improves the Go/Nogo reaction time, but not the simple reaction time. Brain Res Cogn Brain Res 22, 257–264 (2005). 15653298 10.1016/j.cogbrainres.2004.09.003

[b53] Vertopoulos E., Tsolakis C. & Remoundou M. A preliminary study of visual memory and rule detection in fencing: a comparative study. Biology of Exercise 6, 37–45 (2010).

[b54] Unsal A. & Segalowitz S. J. Sources of P300 attenuation after head injury: single-trial amplitude, latency jitter, and EEG power. Psychophysiology 32, 249–256 (1995). 7784533 10.1111/j.1469-8986.1995.tb02953.x

[b55] Walhovd K. B., Rosquist H. & Fjell A. M. P300 amplitude age reductions are not caused by latency jitter. Psychophysiology 45, 545–553 (2008). 18346042 10.1111/j.1469-8986.2008.00661.x

[b56] Rousselet G. A., Husk J. S., Bennett P. J. & Sekuler A. B. Single-trial EEG dynamics of object and face visual processing. Neuroimage 36, 843–862 (2007). 17475510 10.1016/j.neuroimage.2007.02.052

[b57] Ford J. M., White P., Lim K. O. & Pfefferbaum A. Schizophrenics have fewer and smaller P300s: a single-trial analysis. Biol Psychiatry 35, 96–103 (1994). 8167215 10.1016/0006-3223(94)91198-3

[b58] Zhang D., Luo W. & Luo Y. Single-trial ERP evidence for the three-stage scheme of facial expression processing. Sci China Life Sci 56, 835–847 (2013). 23904339 10.1007/s11427-013-4527-8

[b59] Jaśkowski P. & Verleger R. Amplitudes and latencies of single-trial ERP’s estimated by a maximum-likelihood method. IEEE Trans Biomed Eng 46, 987–993 (1999). 10431464 10.1109/10.775409

[b60] Spencer K. M. Averaging, detection, and classification of single-trial ERPs. In Event-Related Potentials: a Method Handbook (ed. Handy T. C.) (The MIT Press, London, 2005).

[b61] Thomalla G. *et al.* Costs of control: decreased motor cortex engagement during a Go/NoGo task in Tourette’s syndrome. Brain 137, 122–136 (2014). 24176975 10.1093/brain/awt288

[b62] Kamijo K. *et al.* The association of childhood obesity to neuroelectric indices of inhibition. Psychophysiology 49, 1361–1371 (2012). 22913478 10.1111/j.1469-8986.2012.01459.x

[b63] Katz R. *et al.* Cognitive control in late-life depression: response inhibition deficits and dysfunction of the anterior cingulate cortex. Am J Geriatr Psychiatry 18, 1017–1025 (2010). 20808083 10.1097/JGP.0b013e3181d695f2 PMC3770530

[b64] Rentrop M. *et al.* Temporal variability and spatial diffusion of the N2 event-related potential in high-functioning patients with schizophrenia. Schizophr Res 131, 206–213 (2011). 21745725 10.1016/j.schres.2011.06.020

[b65] Beste C., Willemssen R., Saft C. & Falkenstein M. Response inhibition subprocesses and dopaminergic pathways: basal ganglia disease effects. Neuropsychologia 48, 366–373 (2010). 19782093 10.1016/j.neuropsychologia.2009.09.023

[b66] Kok A. On the utility of P3 amplitude as a measure of processing capacity. Psychophysiology 38, 557–577 (2001). 11352145 10.1017/s0048577201990559

