# Supplementary material for: Enhanced response inhibition in experienced fencers
Source: Sci Rep. 2015 Nov 6;5:16282. doi: 10.1038/srep16282 (PMC4635338; doi:10.1038/srep16282)
Supplement: Supplementary Information [file srep16282-s1.doc]

# Enhanced response inhibition in experienced fencers

Dandan Zhang^1, #^, Haiyan Ding^2, #^, Xiaochun Wang^3^, *, Changzhu Qi^4^, *, Yuejia Luo^1^

1. Institute of Affective and Social Neuroscience, Shenzhen University, Shenzhen, 518060, China

2. Center for Biomedical Imaging Research, Department of Biomedical Engineering, Tsinghua University, Beijing, 100084, China

3. School of Kinesiology, Shanghai University of Sport, Shanghai, 200438, China

4. Research Center of Sport Psychology, Wuhan Sports University, Wuhan, 430079, China

# These authors contributed equally to this work.

* Correspondence should be sent to Xiaochun Wang (wangxiaochun@sus.edu.cn; Phone: 86-21-51253226) and Changzhu Qi (qicz@qq.com; Phone: 86-27-87191668).

# Additional results

## Accuracy rate (ACC)

The main effect of group was significant (*F*(1,50) = 6.08; *p* = 0.017;
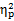
 = 0.108): the ACC in the fencers (0.705 ± 0.017) was larger than that in the non-fencers (0.647 ± 0.017). The main effect of response assignment was also significant (*F*(1,50) = 670; *p* < 0.001;
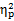
 = 0.931): the ACC in the Go condition (0.986 ± 0.003) was much larger than that in the Nogo condition (0.366 ± 0.024).

## Reaction time (RT)

The main effect of response assignment was significant (*F*(1,50) = 448; *p* < 0.001;
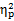
 = 0.900): the RT in the Go condition (443 ± 8.0 ms) was much shorter than that in the Nogo condition (1047 ± 27 ms).

## Average measures of the N2

The main effect of group was significant on the peak amplitude of the average N2 (*F*(1,50) = 4.55; *p* = 0.038;
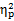
 = 0.083): the N2 amplitude in the fencers (-1.62 ± 0.33 μV) was larger than that in the non-fencers (-0.63 ± 0.33 μV). The main effect of response assignment was also significant (*F*(1,50) = 73.2; *p* < 0.001;
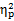
 = 0.594): the N2 amplitude in the Nogo condition (-3.02 ± 0.32 μV) was larger than that in the Go condition (0.78 ± 0.32 μV).

The main effect of response assignment was significant on the peak latency of the average N2 (*F*(1,50) = 46.5; *p* < 0.001;
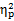
 = 0.482). The N2 latency in the Nogo condition (297 ± 5.72 ms) was longer than that in the Go condition (240 ± 5.52 ms).

## Single-trial measures of the N2

The main effect of response assignment was significant on the standard deviation of the N2 latency (*F*(1,50) = 32.2; *p* < 0.001;
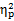
 = 0.392): the Go stimuli elicited a larger latency jitter (standard deviation of latency = 25.6 ± 0.39 ms) than did the Nogo stimuli (22.7 ± 0.33 μV).

## Average measures of the P3

The main effect of group was significant on the peak amplitude of the average P3 (*F*(1,50) = 4.26; *p* = 0.044;
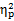
 = 0.078): the P3 amplitude in the non-fencers (8.70 ± 0.45 μV) was larger than that in the fencers (7.38 ± 0.45 μV). The main effect of response assignment was also significant (*F*(1,50) = 48.2; *p* < 0.001;
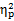
 = 0.491): the P3 amplitude in the Nogo condition (9.34 ± 0.46 μV) was larger than that in the Go condition (6.74 ± 0.26 μV).

The main effect of electrode site was significant on the peak amplitude of the average P3 (*F*(2,102) = 24.8; *p* < 0.001;
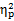
 = 0.327): the P3 amplitude at Fz (4.99 ± 0.29 μV) was smaller than that in the Cz (7.42 ± 0.36 μV) and Pz (7.35 ± 0.30 μV) (*p*s < 0.001). The main effect of response assignment was also significant (*F*(1,51) = 40.0; *p* < 0.001;
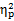
 = 0.439): the P3 amplitude in the Nogo condition (7.64 ± 0.32 μV) was larger than that in the Go condition (5.53 ± 0.24 μV).

The main effect of response assignment was significant on the peak latency of the average P3 (*F*(1,50) = 214; *p* < 0.001;
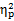
 = 0.811). The P3 latency in the Nogo condition (504 ± 8.38 ms) was much longer than that in the Go condition (355 ± 7.02 ms).

## Single-trial measures of the P3

The main effect of response assignment was significant on the mean of the P3 amplitude (*F*(1,50) = 39.5; *p* < 0.001;
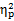
 = 0.441): the P3 amplitude in the Nogo condition (12.5 ± 0.62 μV) was larger than that in the Go condition (9.16 ± 0.35 μV).
